# A single-layer, wideband and angularly stable metasurface based polarization converter for linear-to-linear cross-polarization conversion

**DOI:** 10.1371/journal.pone.0280469

**Published:** 2023-01-20

**Authors:** Aamir Rashid, Mudassir Murtaza, Syed Azhar Ali Zaidi, Hammad Zaki, Farooq A. Tahir

**Affiliations:** 1 Electronics Engineering Department, Faculty of Electronics and Electrical Engineering, University of Engineering and Technology, Taxila, Pakistan; 2 Research Institute of Microwave and Millimetre-Wave Studies, National University of Sciences and Technology (NUST), Islamabad, Pakistan; Universiti Brunei Darussalam, BRUNEI DARUSSALAM

## Abstract

In this article, a single-layer metasurface based reflector design is proposed for linear-to-linear cross-polarization conversion in microwave frequency range. The unit-cell of the proposed design consists of triple-arrow resonant design printed on a grounded FR4 substrate. Excellent cross-conversion is achieved over a broad frequency range (8.0–18.50 GHz) with polarization conversion efficiency higher than 90%. The proposed design has a large fractional bandwidth (FBW) of 80% due to three resonances occurring in the band. The polarization response is angularly stable with respect to oblique incidences with incidence angles up to 45°. The proposed design has been fabricated and experimentally validated. The measurement results are in good agreement with the simulation results.

## 1. Introduction

Metasurfaces are two-dimensional (2-D) metamaterials that find usage in various wireless applications [1, 2]. By definition, a metasurface is a planar structure compromised of subwavelength (thickness smaller than λ/10) resonators arranged in a periodic grid. Metasurfaces on coupling with free space Electromagnetic (EM) waves, induce resonances at desired frequencies at the unit-cell level. Therefore, by judiciously designing the unit-cell one can modify the wave parameters e.g., polarization, of the scattered wave. Polarization conversion (PC) is often required in communications and radar applications to improve radio-link efficiency. Conventional PC is achieved by means of crystals functioning on the principle of Faraday’s effect, but these devices tend to be bulky, narrowband, and costly. Metasurfaces on the other hand can be fabricated on very thin substrates using everyday printed circuit technology at a very low cost. Moreover, the specifications can easily be controlled by applying antenna and microwave design techniques e.g., bandwidth enhancement, impedance matching, beam control and tunability etc. [1, 3, 4].

In the literature, there are numerous designs that have achieved the polarization conversion using metasurfaces in microwave and optical regimes. Feng et al [5] has proposed a wide-band polarization rotator for radio waves (2–3.5GHz) under normal incidence. But the design has a poor polarization conversion ratio (PCR) and suffers from poor angular stability. Chen et al [6] have proposed a design based on arrow-shaped linear resonators for cross-polarization conversion (CPC) over the wide-band from 6-24GHz. But their design achieves above 90% PCR only over a small part of the band. Moreover, the angular stability of the design has not been investigated. Some other designs [7–11], have also proposed designs that can achieve a higher bandwidth by means of multiple resonances but simultaneously achieving a polarization conversion efficiency of greater than 90% remains a big design challenge.

Linear cross-polarization conversion is usually achieved using anisotropic resonators [12–20] in the metasurface design. Anisotropic geometry leads to cross-coupling between electric and magnetic fields components that results in linear-linear cross conversion. Again, due to inherently band limited nature of these resonances, a working multi-band design that can operate over many microwave bands remains a huge design challenge. Recent studies have demonstrated the design and validation of coding metasurfaces for the conversion of linear to circular multi-beam radiation [21, 22]. In this article we present a simple, single-layer metasurface design that achieves cross-conversion for horizontally or vertically polarized incident wave in the reflection mode. Polarization conversion with a conversion efficiency of greater than 90% is achieved over frequency band ranging from 8 to 18.5 GHz. With fractional bandwidth of 80% this design achieves one of the largest operation band in this frequency regime with the angular stability of upto 45°. This article is organized into four sections. Section 1 lays out the literature review and the design challenge. Section 2 details the design and analysis of the reflector metasurface. Subsection 2.1 provides the details of the geometry and dimensions of the unit-cell. Theory of polarization conversion is discussed in subsection 2.2. Subsections 2.3 and 2.4 provide the analysis of the design functionality based on field component analysis along symmetry axes and surface current distribution. The fabrication and measurement details are discussed in Section 3. Section 4 provides the concluding remarks.

## 2. Design and analysis

### 2.1. Unit-cell design

**Fig 1:**
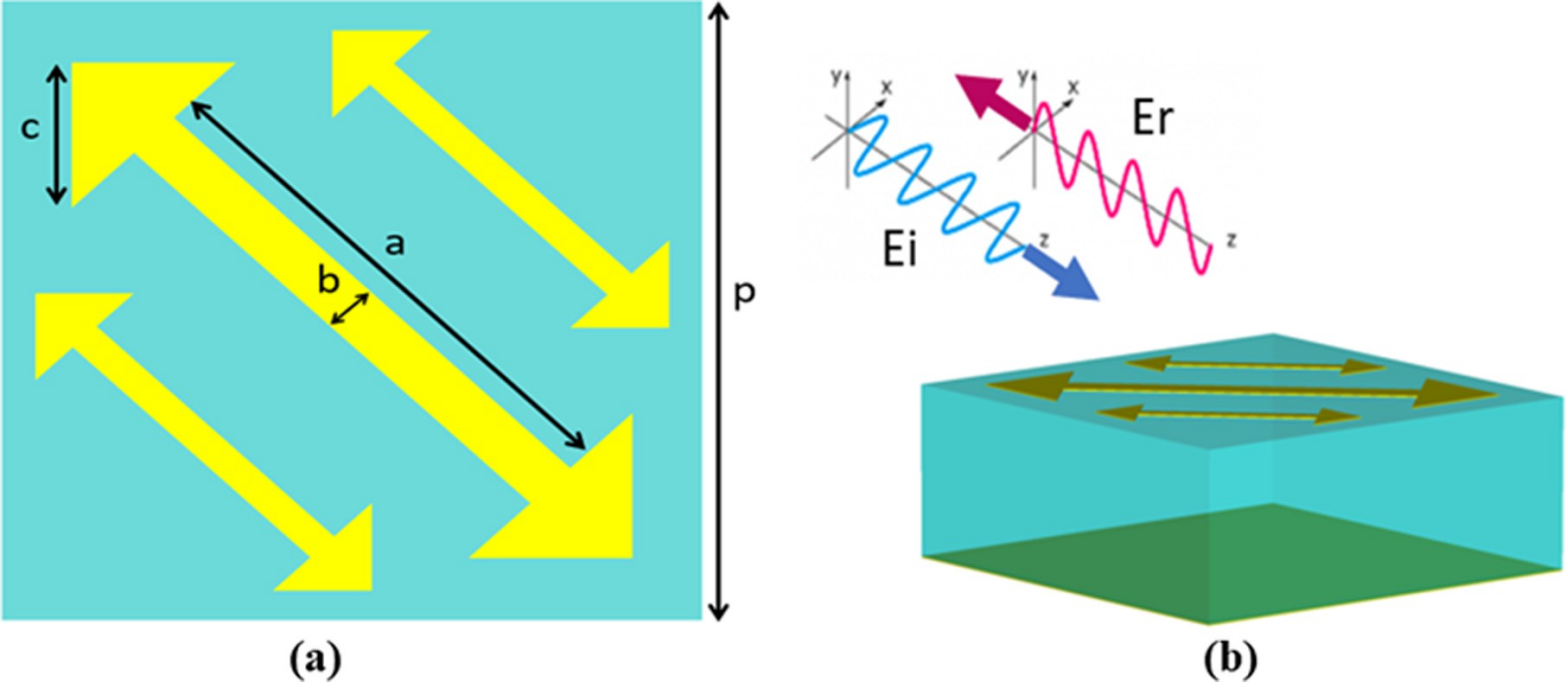
Unit cell schematic design (a) top view (b) 3D view.

The schematic view of a proposed CMR unit cell is presented in **Fig 1**. The unit cell consists of two metallic layers which are separated by FR4 substrate (dielectric constant *ɛ*_*r*_ *= 4*.*4*, and loss tangent *tanδ* = 0.025) of thickness 2.4mm. The FR4 substrate is chosen because it is readily available and low cost, therefore considerably reducing the fabrication cost of the design. The top metallic layer comprises of three arrow-shaped resonators. The bottom layer consists of a metallic ground to prevent transmission. The metallic layers are made up of copper material with conductivity 5.8×10^7^ S/m and thickness of 35 microns. **Fig 1A** shows the optimized parameters of unit-cell design, with labelled dimensions given as: p = 5 mm, a = 3.9 mm, b = 0.4 mm, c = 1.242 mm, α = 0.515, β = 1.5. α is the scaling factor of the smaller side arrows with respect to the center arrow where ±β indicates their displacement with respect to center of the main arrow. The numerical simulation of the metasurface design is performed using CST Microwave Studio® (CST-MW). The simulation of the unit-cell is performed using periodic boundary conditions and Flouqet mode excitation to approximate a large-sized periodic array geometry under plane-wave incidence. The 3D schematic view of a unit cell is depicted in **Fig 1B**.

### 2.2. Analysis based on Jones reflection matrix

The theory of polarization conversion is characterized in terms of Jones transmission and reflection matrices. For a reflector design, transmission is completely zero therefore solely incident and reflected fields are considered. The wave polarization is expressed in terms of its orthogonal components and a comparison between co- and cross-components of incident and reflected fields is used to determine the extent of polarization conversion achieved by the design. The cross-conversion is primarily due to the anisotropic geometry of the unit-cell which interacts with the two orthogonal components differently. Consider a vertically polarized incident wave traveling along +*z* axis, impinging on the metasurface reflector under normal incidence. The electric field vector of the wave is expressed by Eq (1).


$$\vec{E^{i}}=\left|\vec{E_{y}^{i}}\right|e^{-j\varphi_{i}}\vec{e_{y}}$$ (1)

The superscript *i* indicates the incident field whereas the subscript *y* is the vector component label. *φ*_*i*_ represents the phase angle of the field phasor. $\vec{e_{y}}$ is the unit vector along *y*-direction. After reflection from the interface, the electric field vector may get rotated in the *x-y* plane therefore both orthogonal components are required for full description of the reflected wave as in Eq 2A. These component fields can in turn be expressed in terms of complex reflection coefficients as in Eq 2B.


$$\vec{E^{r}}=\left|\vec{E_{x}^{r}}\right|e^{-j\varphi_{x}}\vec{e_{x}}+\left|\vec{E_{y}^{r}}\right|e^{-j\varphi_{y}}\vec{e_{y}}$$ (2a)

$$\vec{E^{r}}=\left|\vec{E_{y}^{i}}\right|(R_{xy}e^{-j\varphi_{x}}\vec{e_{x}}+R_{yy}e^{-j\varphi_{y}}\vec{e_{y}})$$ (2b)

In the above equations, superscript *r* denotes the reflected field whereas the subscripts *x* and *y* are field component labels. The general relation between linearly polarized incidence and reflected fields can be summarized in the form of Jones reflection matrix comprised of four co- and cross-reflection coefficients as given in Eq 3.


$$\left[\begin{array}{c} E_{xr}\\ E_{yr} \end{array}\right]=\left[\begin{array}{cc} R_{xx} & R_{xy}\\ R_{yx} & R_{yy} \end{array}\right]\left[\begin{array}{c} E_{xi}\\ E_{yi} \end{array}\right]=R_{lin}\left[\begin{array}{c} E_{xi}\\ E_{yi} \end{array}\right]$$ (3)

*R*_*lin*_ is called Jones Reflection Matrix characterizing wave reflection phenomenon for linearly polarized waves. *R*_*xx*_ and *R*_*yy*_ are co-polarization reflection coefficients defined as the ratio of the magnitudes of horizontal reflected to horizontal incident field (*R*_*xx*_ *= |E*_*xr*_*|/|E*_*xi*_*|*) and vertical reflected to vertical incident field (*R*_*yy*_ *= |E*_*yr*_*|/|E*_*yi*_|). Similarly, *R*_*xy*_ and *R*_*yx*_ are cross-polarization reflection coefficients relating the cross-field components i.e., *R*_*xy*_ *= |E*_*xr*_*|/|E*_*yi*_*|* and *R*_*yx*_ *= |E*_*yr*_*|/|E*_*xi*_|.

### 2.3. Design evolution

**Fig 2:**
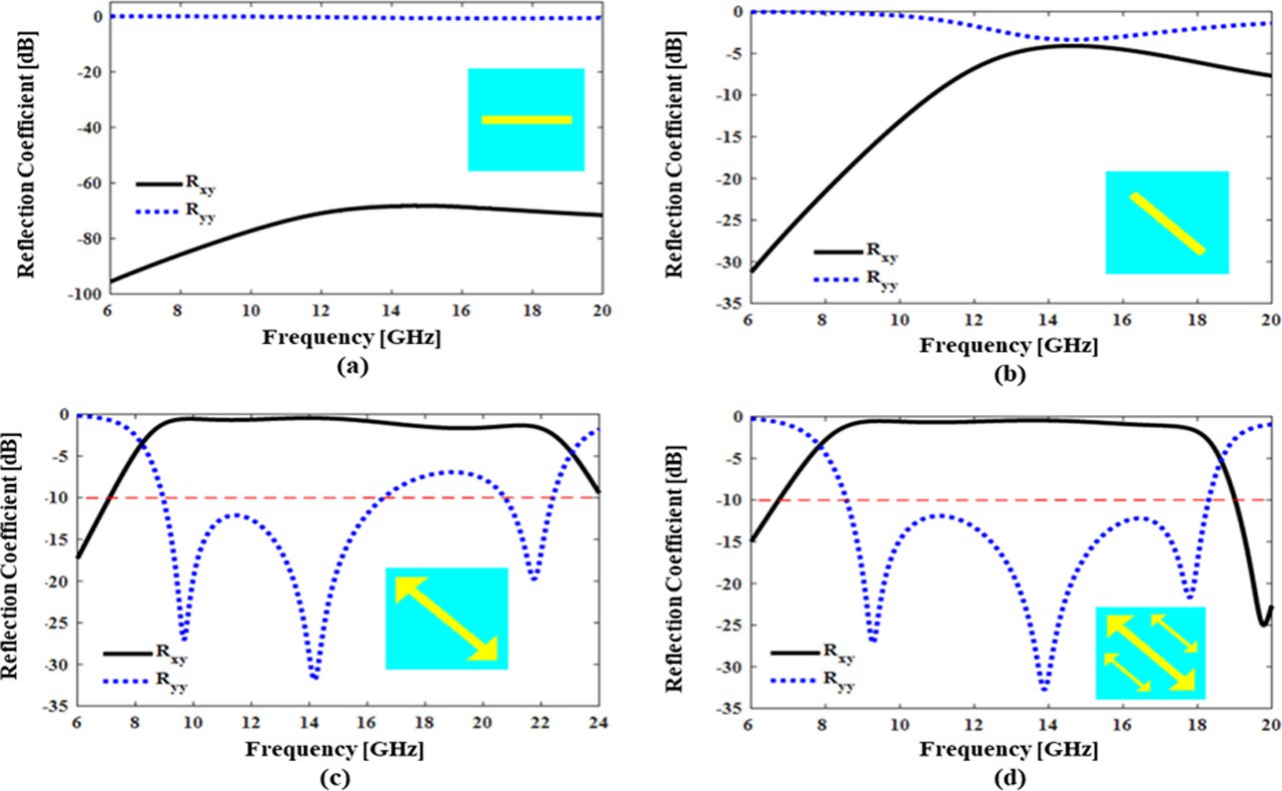
Co- and Cross polarized reflection coefficient for (a) horizontal line (b) diagonal line (c) single arrow shaped split ring resonators (d) triple- arrows shaped split ring resonators.

The design of the unit-cell has been performed in multiple steps. Initially we consider a simple metallic line-resonator lying horizontally as shown in **Fig 2A**. The reflection response of this design is studied for *y*-polarized incident wave and reflection coefficient results are shown in **Fig 2A**. It is apparent that co-polarization coefficient *R*_*yy*_ is maximum and cross-polarization coefficient *R*_*xy*_ = 0 over the entire band. The reason there is no cross-conversion is due to the lack of anisotropy in the design. A *y*-polarized field when resolved in terms of orthogonal components along diagonal axes (±45° w.r.t *y*-axis), both components experience similar design symmetry resulting in reflection with no phase difference between the two, therefore no polarization conversion is achieved. For polarization conversion, anisotropy in structure is needed, consequently, in step 2 we replace a horizontal line with a diagonal line to break this isotropy. It can be seen in **Fig 2B**, that the diagonally oriented resonator increases the cross-polarization reflection and *R*_*xy*_ approaches -4dB at 14.58GHz. But still this design cannot achieve strong cross-polarization conversion. Therefore, in step 3, the line resonator is replaced with an arrow-shaped resonator to enhance the resonance effects. The results in **Fig 2C** show that *R*_*xy*_ now approaches -3 dB and *R*_*yy*_ approaches -5dB in 8.3GHz to 23.01GHz band. In step 4 two additional arrow-shaped resonators are included in the design to introduce additional resonances. The final and optimized design increases the polarization conversion efficiency by suppressing the co-polarization reflection coefficient *R*_*yy*_ below -10dB and improving cross-polarization reflection coefficient *R*_*xy*_ above -1dB in frequency regime of 8.0GHz to 18.50GHz as shown in **Fig 2D**. The strong cross-conversion response of this design is due to dissimilar electric and magnetic responses to the components of the incident fields along the two diagonal axes. The design has a predominantly inductive response to incident field component along +45° due to current path along the arrow lengths and has a capacitive response to component along -45° due to the gap between these three arrows.

**Fig 3:**
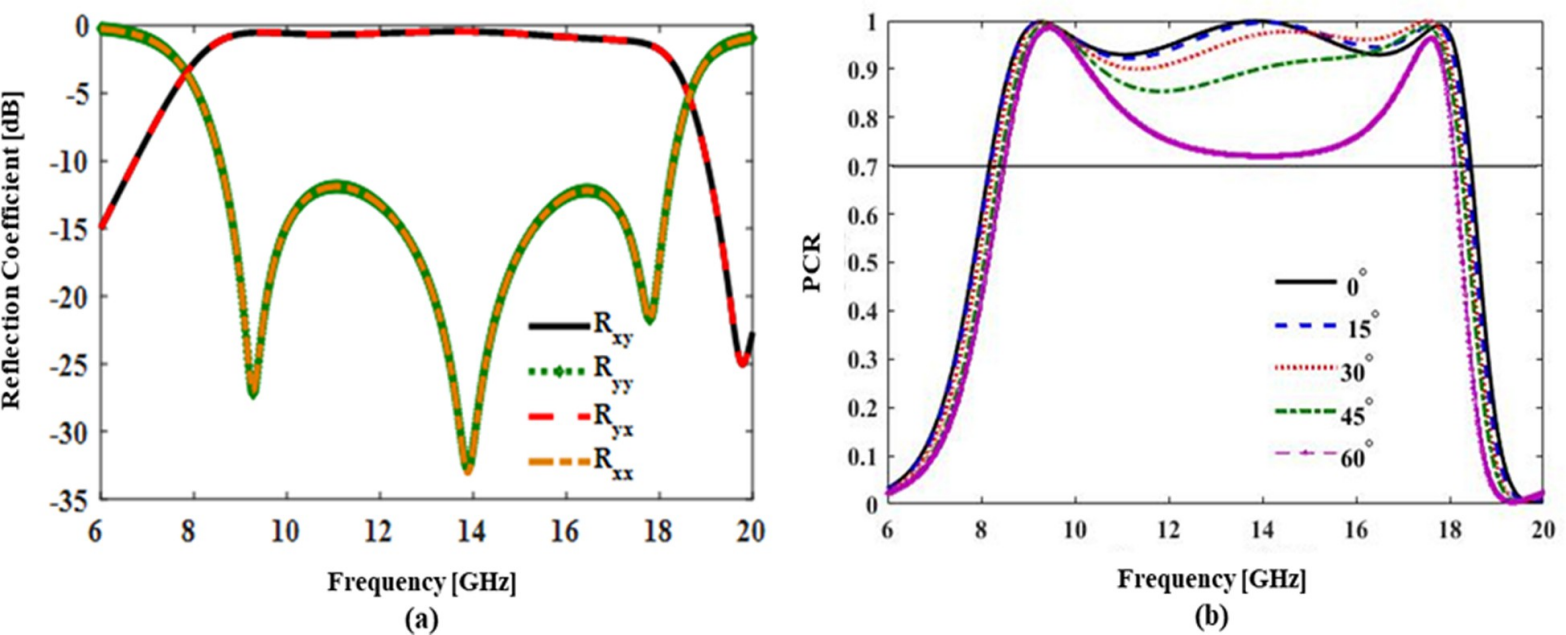
(a) Magnitude of Cross- and Co-polarized reflection coefficients for both *x*- and *y*-polarized incident waves (b) Polarization Conversion Ratio (PCR) results under normal and oblique incidences.

The magnitude of co- and cross-polarization reflection coefficients under normal incidence for both *x*- and *y*-polarized incident waves are shown in **Fig 3A.** It can be seen clearly in **Fig 3A** that the co-polarization reflection coefficients R_yy_ and R_xx_ are less than -10dB and cross-polarization reflection coefficients R_xy_ and R_yx_ are greater than -1dB over a wide range of frequency band (8.0GHz to 18.50GHz). Three plasmonic resonances occurring at 9.28GHz, 13.91GHz, and 17.80GHz are also depicted in **Fig 3A** where the magnitude of co-polarization reflection coefficient is minimum at -27.16 dB, -32.60 dB and -21.65 dB respectively. The cross-polarization reflection coefficient magnitude at these resonances is nearly equal to 0dB.

To find the efficiency of polarization conversion we use a metric called polarization conversion ratio (PCR) which is defined as *PCR = |R*_*xy*_*|*^*2*^ */ (|R*_*xy*_*|*^*2*^ *+ |R*_*yy*_*|*^*2*^*)*. It is a measure of power in the reflected cross-polarization component to the total reflected power. For ideal cross conversion PCR = 1 or conversion efficiency of 100%. **Fig 3B** plots the PCR results for normal and oblique incidences of up to 60°. It can be seen from the normal incidence curve (theta = 0°) that the conversion efficiency is 100% at three plasmonic resonances (9.28GHz, 13.91GHz, and 17.80GHz) and the efficiency is greater than 90% over a wide band of frequency (8.0–18.50GHz). The plot also shows that oblique incidence response is stable at above 90% efficiency upto an angle of 30° and even at 45° the conversion efficiency is greater than 86% over the entire band. Angular stability of up to 45° is a reasonable result for most applications therefore we can state that our design is angular stable. The response for 60° incidence angle is also plotted in the same figure. The conversion efficiency significantly deteriorates as compared to smaller angles but still the efficiency is greater than 70% mark over the entire band.

### 2.4. Analysis based on structural symmetry

**Fig 4:**
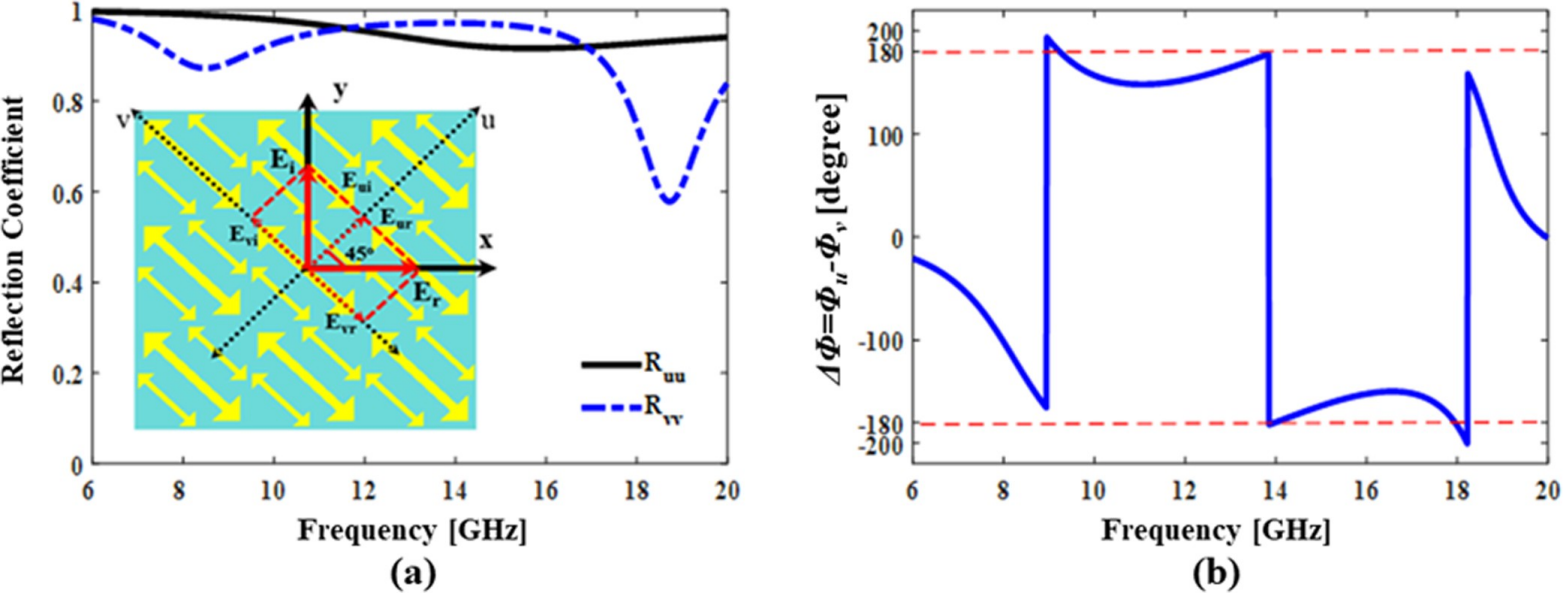
(a) Magnitude (in linear scale) of co-polarized reflection coefficients (R_uu_, R_vv_) for u- and v-component waves (b) Phase difference (in degrees) between R_uu_ and R_vv_.

Structural symmetry analysis, also known as *UV*-analysis is used to better understand the response of a proposed structure. In this analysis we choose two symmetry axes and label them as *u* and *v* axes as shown in **Fig 4**.

All fields (incident and reflected) are resolved in terms of components along these axes directions. For example, *y*-polarized incident field is split in terms of *u*- and *v*-components as shown in **Fig 4A**. Eq **4A** and **4B** depicts the incident electric field and Eq **5A–5E**) indicates reflected electric field when written in terms of *u* and *v* components.

$$\underline{E_{i}}=\hat{e}E_{yi}=\hat{u}E_{ui}+\hat{v}E_{vi}=\left[\begin{array}{c} E_{ui}\\ E_{vi} \end{array}\right]$$ (4a)

$$\underline{E_{i}}=\left[\begin{array}{c} E_{yi}/\sqrt{2}\\ E_{yi}/\sqrt{2} \end{array}\right]=E_{yi}/\sqrt{2}(\hat{u}+\hat{v})$$ (4b)

$$\underline{E_{r}}=\hat{u}E_{ur}+\hat{v}E_{vr}=\left[\begin{array}{c} E_{ur}\\ E_{vr} \end{array}\right]=\left[\begin{array}{cc} R_{uu} & R_{uv}\\ R_{vu} & R_{vv} \end{array}\right]\left[\begin{array}{c} E_{ui}\\ E_{vi} \end{array}\right]$$ (5a)

Here, *E*_*ui*_ and *E*_*vi*_ represent incident electric field components and *E*_*ur*_ and *E*_*vr*_ represent reflected electric field components along *u*- and *v*- directions, respectively. For linear cross-conversion |*R*_*uv*_| = |*R*_*vu*_| = 0, Therefore

$$\underline{E_{r}}=\hat{u}E_{ur}+\hat{v}E_{vr}=\hat{u}R_{uu}E_{ui}+\hat{v}R_{vv}E_{vi}$$ (5b)

Where $R_{uu}=\left|R_{uu}\right|e^{j\varphi_{uu}}$ and $R_{vv}=\left|R_{vv}\right|e^{j\varphi_{vv}}$. If |R_uu_| = |R_vv_| and the phase difference *φ*_*uu*_*− φ*_*vv*_ = Δφ, R_vv_ can be expressed as *R*_*vv*_ = *R*_*uu*_^*ej*Δ*φ*^

$$\underline{E_{r}}=\left[\begin{array}{c} E_{ur}\\ E_{vr} \end{array}\right]=\left[\begin{array}{cc} R_{uu} & 0\\ 0 & R_{uu}e^{j\Delta \varphi } \end{array}\right]\left[\begin{array}{c} E_{yi}/\sqrt{2}\\ E_{yi}/\sqrt{2} \end{array}\right]$$ (5c)

$$\underline{E_{r}}=R_{uu}E_{yi}/\sqrt{2}(\hat{u}+\hat{v}e^{j\Delta \varphi })$$ (5d)

Additionally, if |R_uu_| = |R_vv_| = 1 and Δφ **=** ±180°, the reflected field is 90° rotated version of the incident field as given by the Eq 5E

$$\underline{E_{r}}=E_{yi}/\sqrt{2}(\hat{u}-\hat{v})$$ (5e)

**Fig 4A** also shows that the magnitude of both co-polarization reflection coefficients (along symmetry axes i.e., *R*_*uu*_ and *R*_*vv*_) is nearly equal to 1 for *u*- and *v*-polarized incident waves over desired band of 8GHz to 18.5GHz. The magnitude of cross-polarization reflection coefficients (*R*_*uv*_ and *R*_*vu*_) is much smaller and nearly zero. The phase difference Δφ (*φ*_*uu -*_ *φ*_*vv*_) between co-polarization reflection coefficients (*φ*_*uu*_ is the phase of *R*_*uu*_ and *φ*_*vv*_ is the phase of *R*_*vv*_) is nearly ±180° over a wideband 8GHz to 18.5GHz as show in **Fig 4B**. From the field Eq **5A–5E**, the condition for cross-polarization conversion is fulfilled when the magnitude of cross-polarization reflection coefficients is equal to zero (|*R*_*uv*_*| = |R*_*vu*_*| = 0*), co-polarization reflection coefficients is equal to 1 *(|R*_*uu*_*| = |R*_*vv*_*| = 1*) and the phase difference between the two is 180° (*Δφ* ***=*** *±180*^*o*^). **Fig 4** demonstrates that the proposed design fulfills these requirements for cross-polarization conversion over a wide frequency range.

### 2.5. Analysis based on surface currents

**Fig 5:**
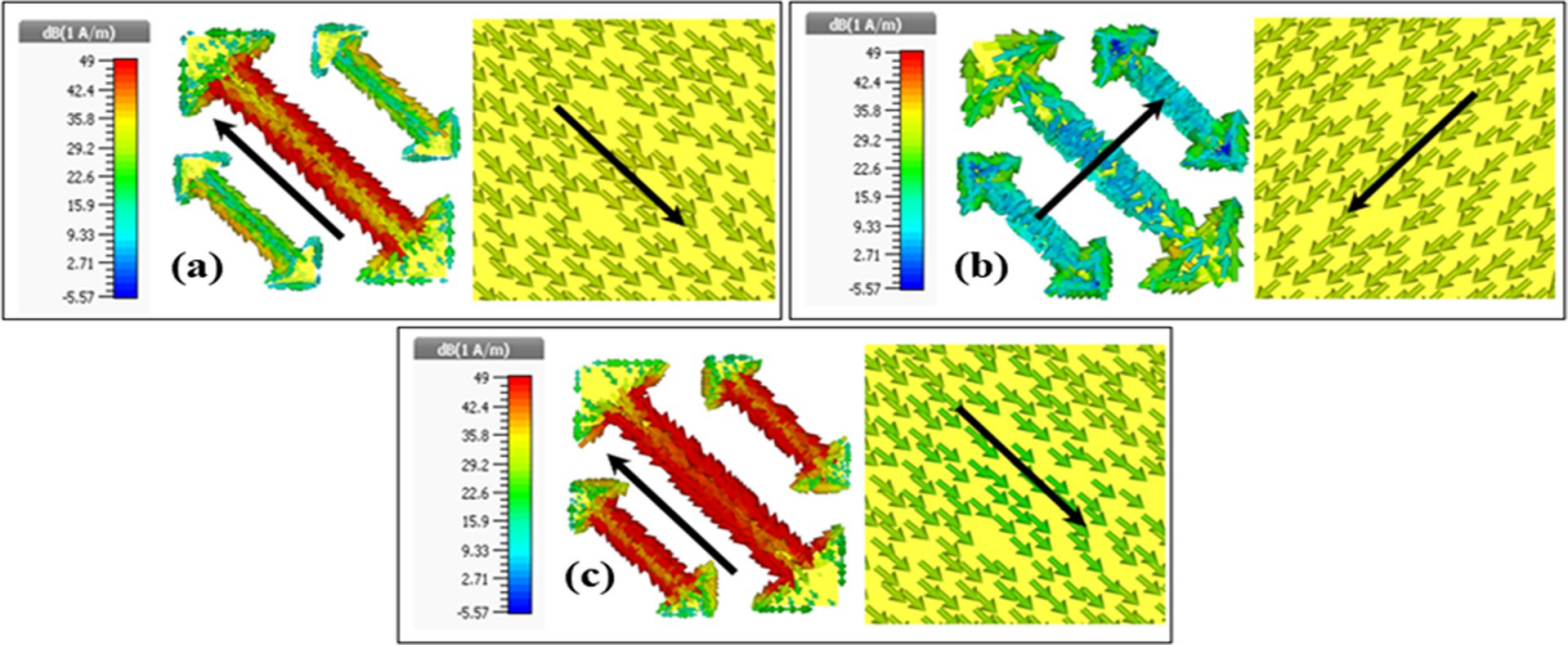
Surface current distributions on top and bottom plane at three different resonance frequencies (a) 9.28 GHz (b) 13.91 GHz (c) 17.80 GHz.

The surface current distribution on the metasurface structure as well as the ground plane of the unit-cell is used to study the physical mechanism for a cross-polarization conversion. **Fig 5A–5C** shows three plasmonic resonances (9.28GHz, 13.91GHz and 17.80GHz) due to coupling between parallel and antiparallel currents on the top layer (pattern layer) and bottom layer (ground) respectively. The anti-parallel currents on top and bottom layers generate strong magnetic resonance. Resonances at 9.28GHz and 17.80GHz depicted in **Fig 5A and 5C** are due to strong currents along outer edges (lengths) of arrows and resonance at 13.91GHz shown in **Fig 5B** is due to the currents along arrow widths.

## 3. Design validation

**Fig 6:**
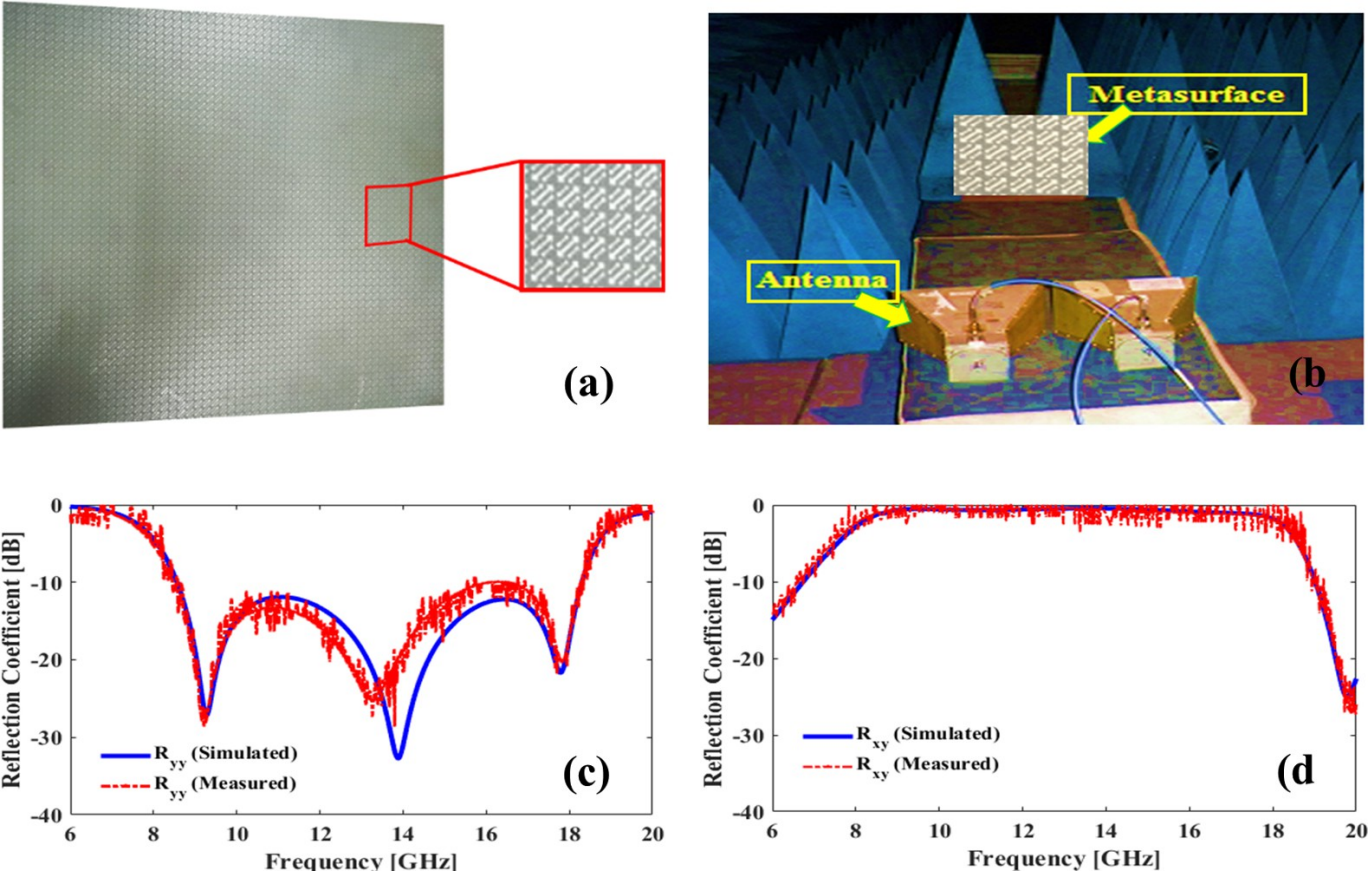
(a) Picture of the fabricated sample. (b) Measurement setup inside an anechoic chamber. (c) simulation and measurement results of co-polarization reflection coefficient *R*_*yy*_. (d) simulation and measurement results of cross-polarization reflection coefficient *R*_*xy*_.

The experimental validation of the simulation results is performed by fabricating our proposed design as 44×44 unit-cell array on a copper backed FR4 substrate of dimensions 305×305 mm^2^. The array dimensions are large enough that periodic array approximation holds. The fabricated design is shown in **Fig 6A**. Wideband double-ridged horn antennas (2–20 GHz) are used as transmitting and receiving antennas in the measurement setup. The measurement of scattering parameters is performed using Agilent Vector Network Analyzer N5232A inside an anechoic chamber. The entire measurement setup is depicted in **Fig 6B**. Orthogonal polarization components are measured by orienting the receiving horn along horizontal or vertical axis. For example, to measure co-polarization reflection coefficient R_yy_ both antennas are oriented vertically i.e., along y-axis. On the other hand, to measure cross-polarization reflection coefficient R_xy_, transmitting antenna is oriented vertically while receiving antenna is oriented horizontally (along x-axis). R_xx_ and R_yx_ can be measured in a similar manner.

**Fig 6C and 6D** compares the measurement and simulation results for *y*-incidence polarization from 6 to 20GHz band. The results shows that there is good agreement between the two results in the entire band. The middle resonance around 14 GHz is a little displaced in the measurement results (**Fig 6C**) but it can be attributed to small fabrication errors in the design. The overall response of the design compares nicely to the expected simulation results. Hence the design response is experimentally validated over the entire band of operation.

**Table 1** presents the performance comparison of our design with some reference designs. The fractional bandwidth of the proposed metasurface is significantly better as compared to other cross-conversion designs. Ref [9, 10] present designs that only work for normal incidence case and have fractional bandwidth (FBW) significantly smaller than our design. The fractional bandwidth of design [13] is 86.9% but the design has poor angular stability. Ref [14, 15] have good angular stability results but these designs are limited by smaller bandwidth and poor polarization efficiency. The design in the present study not only achieves excellent bandwidth response but also great angular stability response.

**Table 1:** Comparison with other cross-polarization conversion metasurface designs.

Designs	Substrate with thickness	Operating Bandwidth (GHz)	PCR	Fractional Bandwidth (FBW) (%)	Angular stability
	(electrical size)				
**Ref. [9]**	F4B-2	9.1–12.9	99%	34.5%	Only for normal incidence wave.
	0.06 λ_o_				
**Ref. [10]**	FR4	9.24–17.64	90%	62.5%	Only for normal incidence wave.
	0.05 λ_o_				
**Ref. [13]**	F4B	14.2–36	90%	86.9%	Angularly stable up-to 20° b/w 14-27GHz only.
	0.067 λ_o_				
**Ref. [14]**	FR4	5–9.7 and 11.2–15	60%	63.9%	Angularly stable up-to 60°.
	0.04 λ_o_				
**Ref. [15]**	FR4	5–10.8	60%	73%	Angularly stable up-to 45°.
	0.04 λ_o_				
**Present Study**	FR4	8–18.5	90%	80%	Angularly stable up-to 45°.
	0.048 λ_o_				

## 4. Conclusion

In conclusion, in this work we have proposed a highly efficient, low-cost, low-profile, and wideband cross-polarization conversion design with good angular stability response. The simulation and measurement results have conclusively shown that the design works as a near perfect cross-converter over a broad frequency regime (8 to 18.5 GHz) for both horizontal and vertical incidence polarizations. This conversion response is further demonstrated to be angularly stable up to incidence angles of 45°. The PCR of greater than 90% is achieved over the entire band (FBW of 80%). The design evolution to achieve these results has been presented in an incremental manner. Moreover, detailed analysis based on fields, symmetry and surface current distribution has been carried out to understand the electromagnetic phenomenon responsible for the cross-polarization conversion. Finally, a comparison with existing single layered designs using similar substrates has been provided.

## Supporting information

S1 File CST Design file “CSTDesign.cst” has been provided as a supporting document which can be used to simulate our design in CST Studio. (CST)
